# Author Correction: Deep transfer learning for reducing health care disparities arising from biomedical data inequality

**DOI:** 10.1038/s41467-020-20480-x

**Published:** 2020-12-18

**Authors:** Yan Gao, Yan Cui

**Affiliations:** 1 Department of Genetics, Genomics and Informatics, University of Tennessee Health Science Center, Memphis, TN 38163 USA; 2 Center for Integrative and Translational Genomics, University of Tennessee Health Science Center, Memphis, TN 38163 USA; 3 Center for Cancer Research, University of Tennessee Health Science Center, Memphis, TN 38163 USA

Correction to: *Nature Communications* 10.1038/s41467-020-18918-3, published online 12 October 2020.

The original version of the Article originally referenced a “Supplementary Table 1” that was missing from the Supplementary Information file. This has now been provided as Supplementary Data 1. Therefore all references to Supplementary Table 1 in the Article have been changed to Supplementary Data 1, as follows:

The second paragraph of the Results section ‘Disparities in machine learning model performance’ read: “For each of the 224 learning tasks (Supplementary Table 1), we performed six machine learning experiments (Table 1) to compare the performance of the three multiethnic machine learning schemes on the AA and EA groups (Fig. 2).” This has now been corrected to: “For each of the 224 learning tasks (Supplementary Data 1), we performed six machine learning experiments (Table 1) to compare the performance of the three multiethnic machine learning schemes on the AA and EA groups (Fig. 2).”

The third paragraph of the Results section ‘Transfer learning for improving machine learning model performance for data-disadvantaged ethnic groups’ read: “We also performed the machine learning experiments on two additional learning tasks that involved either another ethnic group or non-TCGA data: (1) Stomach Adenocarcinoma (STAD)-EAA/EA-PFI-2YR assembled using the TCGA STAD data of EAA and EA patients; and (2) MM-AA/EA-mRNA-OS3YR assembled using the MMRF CoMMpass27 data of AA and EA patients (Supplementary Table 1).” This has now been corrected in the new version to: “We also performed the machine learning experiments on two additional learning tasks that involved either another ethnic group or non-TCGA data: (1) Stomach Adenocarcinoma (STAD)-EAA/EA-PFI-2YR assembled using the TCGA STAD data of EAA and EA patients; and (2) MM-AA/EA-mRNA-OS3YR assembled using the MMRF CoMMpass27 data of AA and EA patients (Supplementary Data 1).”

The legend of Fig. 3 in the original version of the Article reported: “Abbreviations for cancer types are explained in Supplementary Table 1.” This has now been corrected in the new version to: “Abbreviations for cancer types are explained in Supplementary Data 1.”

This has been corrected in both the PDF and HTML versions of the Article.

## Supplementary information

Supplementary Information

