# Supplementary material for: Deep transfer learning for reducing health care disparities arising from biomedical data inequality
Source: Nat Commun. 2020 Oct 12;11:5131. doi: 10.1038/s41467-020-18918-3 (PMC7552387; doi:10.1038/s41467-020-18918-3)
Supplement: Supplementary file 1 — Supplementary Information [file 41467_2020_18918_MOESM1_ESM.pdf]

**Supplementary information**

# Deep transfer learning for reducing health care disparities arising from biomedical data inequality

Yan Gao[1,2] and Yan Cui[1,2,3,*]

[1]Department of Genetics, Genomics and Informatics, [2]Center for Integrative and Translational Genomics, [3]Center for Cancer Research, University of Tennessee Health Science Center, Memphis, TN 38163, USA

*Corresponding author. Email: ycui2@uthsc.edu

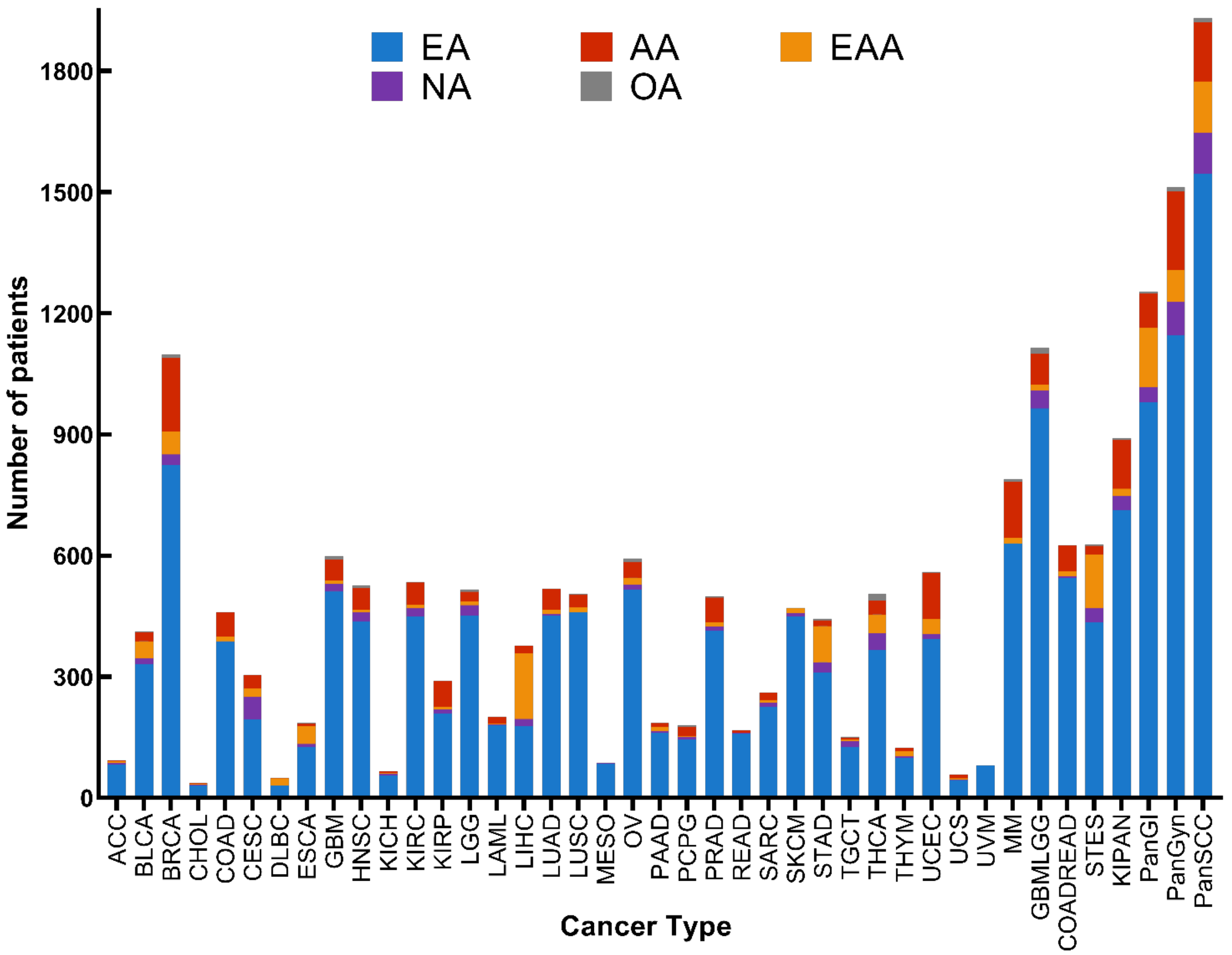


**Supplementary Fig. 1 Ethnic compositions of the TCGA and MMRF CoMMpass cohorts for each cancer type.** The abbreviations used in the figure: TCGA: The cancer genome atlas; AA, African American; EA, European American; EAA, East Asian American; NA, Native American; OA, Other; abbreviations for cancer types are explained in Supplementary Table 1.

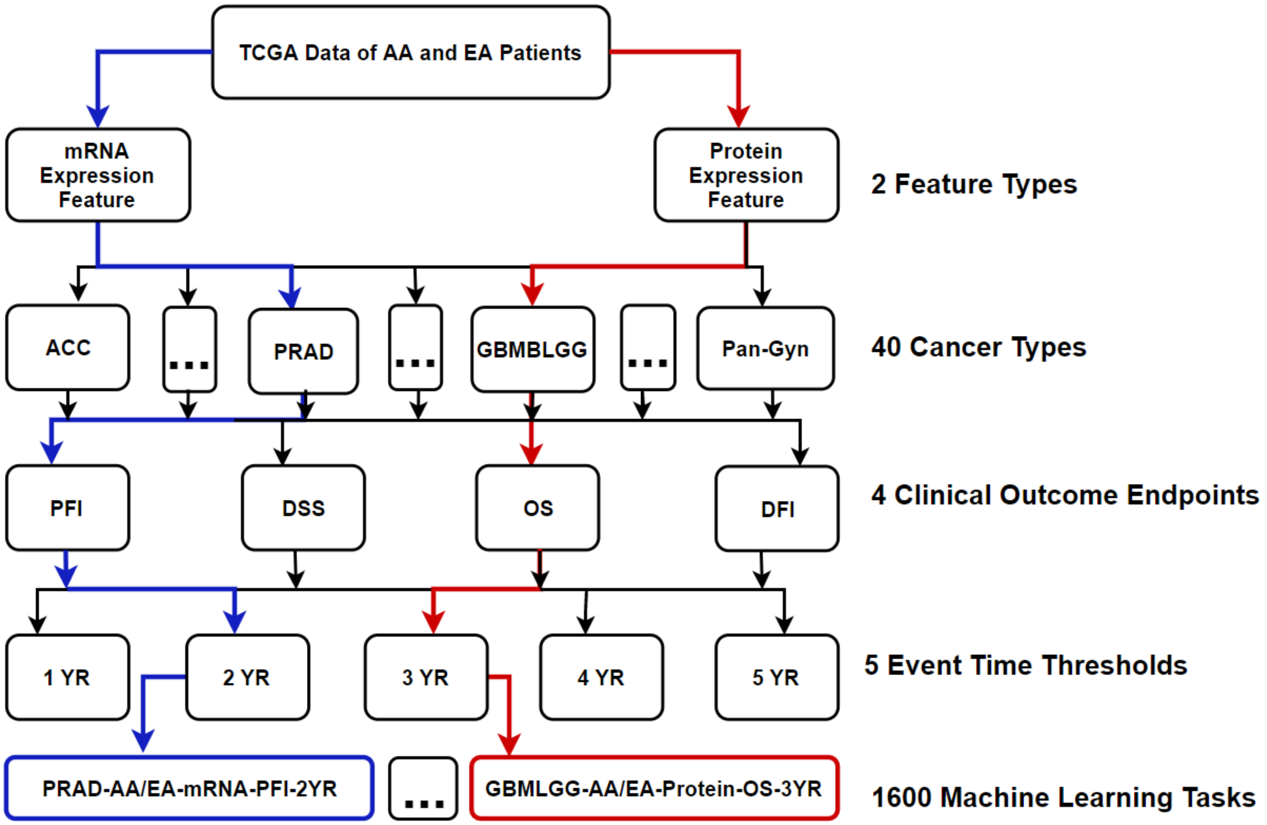


**Supplementary Fig 2 Assembly of machine learning tasks.** The machine learning tasks were assembled using combinations of four factors: feature type, cancer type, clinical outcome endpoint and event time for the clinical outcome endpoints. Each path (e.g. the red path and blue path) represents the assembly of a machine learning task. Abbreviations used in the figure: TCGA: The cancer genome atlas; PFI, progression-free interval; DSS, disease-specific survival; OS, overall survival; DFI, disease-free interval; abbreviations for cancer types are explained in Supplementary Table 1.

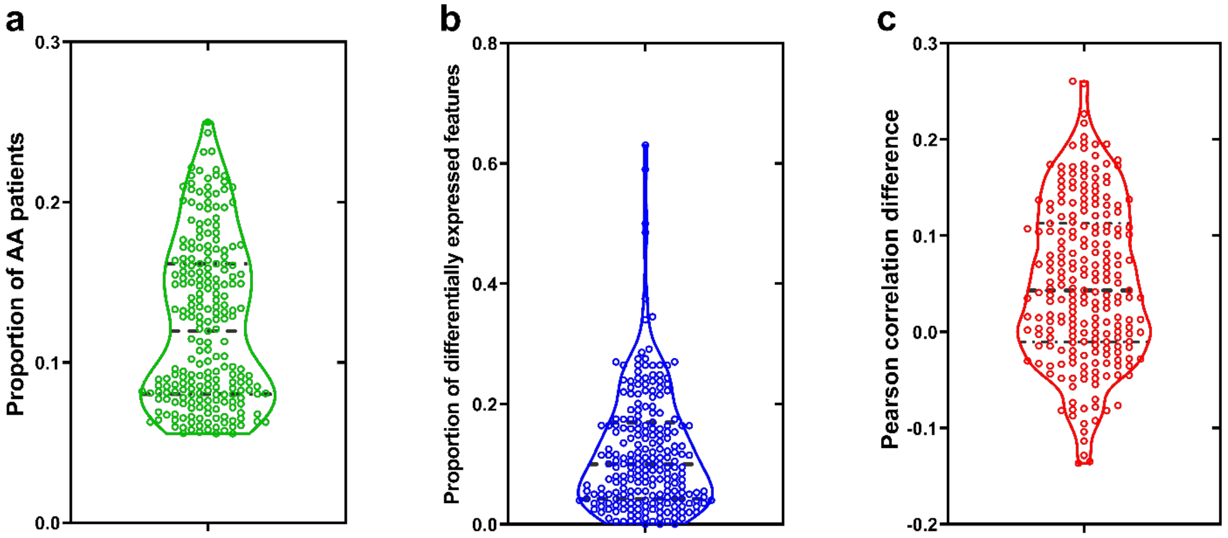


**Supplementary Fig. 3 Assessment of key factors underlying ethnic disparities in machine learning model performance. a** Proportions of AA patients. **b** Proportions of differentially expressed mRNA or protein features between the AA and EA groups. **c** Pearson correlation coefficients between the logistic regression parameters for the AA and EA groups in the 224 learning tasks (Supplementary Table 1). Violin plot elements are: center line, median; lower and upper lines, 25 and 75 percentiles.

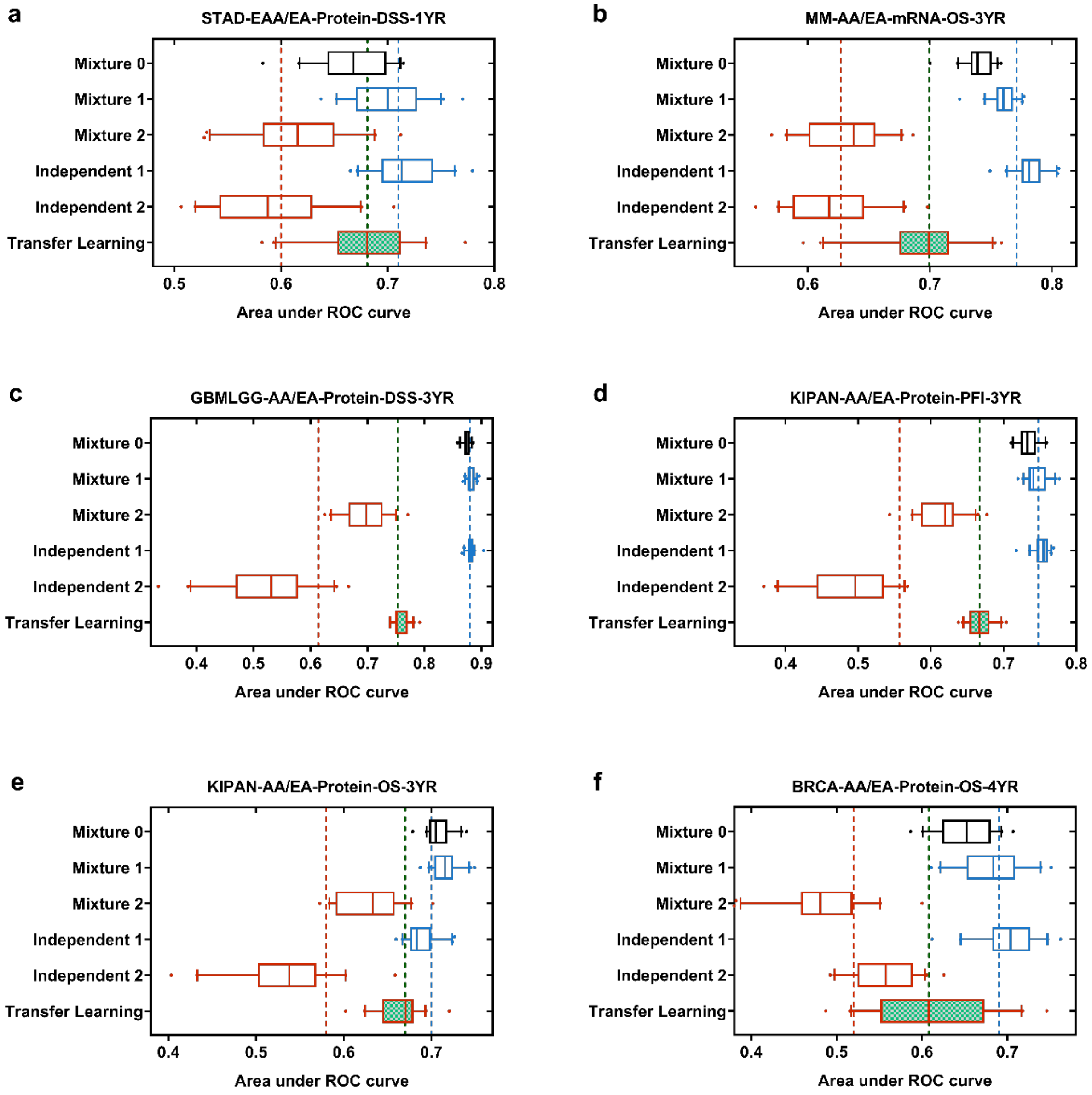


**Supplementary Fig. 4 Comparison of multiethnic machine learning schemes on cancer omics data.** The machine learning tasks are: **a** STAD-EAA/EA-Protein-DSS-1YR, **b** MM-AA/EA-mRMA-OS-3YR, **c** GBMLGG-AA/EA-Protein-DSS-3YR, **d** KIPAN-AA/EA-Protein-PFI-3YR, **e** KIPAN-AA/EA-Protein-OS-3YR, **f** BRCA-AA/EA-Protein-OS-4YR. In each panel, the box plots show the AUROC values for the six experiments (20 independent runs for each experiment). The red, blue and green vertical dash line represents $\overline{AUROC}_{AA}$, $\overline{AUROC}_{EA}$ and $A_{Transfer}$ respectively. Box-plot elements are: center line, median; box limits, 25 and 75 percentiles; whiskers, 10 to 90 percentiles; points, outliers. The machine learning experiments are described in Table 1. Abbreviations for cancer types are explained in Supplementary Table 1.
